# Fungal phytopathogen modulates plant and insect responses to promote its dissemination

**DOI:** 10.1038/s41396-021-01010-z

**Published:** 2021-06-14

**Authors:** Flávia P. Franco, Amanda C. Túler, Diego Z. Gallan, Felipe G. Gonçalves, Arodí P. Favaris, Maria Fernanda G. V. Peñaflor, Walter S. Leal, Daniel S. Moura, José Maurício S. Bento, Marcio C. Silva-Filho

**Affiliations:** 1 Departamento de Genética, Escola Superior de Agricultura Luiz de Queiroz, Universidade de São Paulo, Piracicaba, SP Brazil; 2 Departamento de Entomologia e Acarologia, Escola Superior de Agricultura Luiz de Queiroz, Universidade de São Paulo, Piracicaba, SP Brazil; 3 Departamento de Entomologia, Universidade Federal de Lavras, Lavras, MG Brazil; 4 Department of Molecular and Cellular Biology, University of California, Davis, CA USA; 5 Departamento de Ciências Biológicas, Escola Superior de Agricultura Luiz de Queiroz, Universidade de São Paulo, Piracicaba, SP Brazil

**Keywords:** Molecular ecology, Molecular ecology

## Abstract

Vector-borne plant pathogens often change host traits to manipulate vector behavior in a way that favors their spread. By contrast, infection by opportunistic fungi does not depend on vectors, although damage caused by an herbivore may facilitate infection. Manipulation of hosts and vectors, such as insect herbivores, has not been demonstrated in interactions with fungal pathogens. Herein, we establish a new paradigm for the plant-insect-fungus association in sugarcane. It has long been assumed that *Fusarium verticillioides* is an opportunistic fungus, where it takes advantage of the openings left by *Diatraea saccharalis* caterpillar attack to infect the plant. In this work, we show that volatile emissions from *F. verticillioides* attract *D. saccharalis* caterpillars. Once they become adults, the fungus is transmitted vertically to their offspring, which continues the cycle by inoculating the fungus into healthy plants. Females not carrying the fungus prefer to lay their eggs on fungus-infected plants than mock plants, while females carrying the fungus prefer to lay their eggs on mock plants than fungus-infected plants. Even though the fungus impacts *D. saccharalis* sex behavior, larval weight and reproduction rate, most individuals complete their development. Our data demonstrate that the fungus manipulates both the host plant and insect herbivore across life cycle to promote its infection and dissemination.

## Introduction

Plant-insect-fungus interactions involve plant defense responses that eventually influence insect behavior or pathogen infection [1–3]. Changes in plant metabolites and insect behavior can be explained by the “host manipulation hypothesis”, which states that the pathogen can manipulate the insect vector and/or final host response to guarantee infection and dissemination [4–7]. Phytopathogens can change plant phenotypes, nutritional profiles, and the emission of volatile organic compounds (VOCs) [3], as a strategy to attract vectors and disseminate them [6]. An example is when the phytopathogen infection increases pre-existing host-location cues that are attractive to the vector, but also reduces host nutritional quality, making the vector move to a healthy plants [6]. Microorganisms can also alter insect behavior, for example, when they induce an increase in aggregation pheromone production resulting in the attraction of more individuals and, consequently, infecting more insects [8], when they affect insects’ feeding behavior [9], perception of odors [10, 11] or tastes [12]. An extraordinary example of changes of insect behavior is “zombie-ants” which fungus manipulates the ants to behave like “zombies,” to bite the underside of vegetation and spread the spores on nature [13]. On the other hand, insects can participate in fungal dispersion by spreading spores over long distances [14, 15] and/or facilitating the entry of opportunistic fungi through feeding [16] as can occur in Ambrosia beetles [17]. As a vector, insects can transmit pathogens horizontally (environmental source), vertically (maternal inheritance) or, rarely, inheritance from both parents, or via a mixture of horizontal and vertical transfer [18].

Differently from vector-borne pathogens, opportunistic pathogens presumably do not have an intimate association with the insect. In sugarcane, infection by the fungal phytopathogen *Fusarium verticillioides* usually occurs in association with *Diatraea saccharalis* (F.) caterpillars. Hitherto, it was assumed that the fungus takes advantage of the openings made by the borer to penetrate the sugarcane stalk and infect the plant, resulting in additional crop damage due to Pokkah Boeng disease [19, 20]. In fact, it has been shown that *D. saccharalis* attack induces the production of sugarcane defensive proteins with antifungal activity [21–23]. These proteins affect *F. verticillioides* morphology, causing fungal death, but are not able to affect *Aspergillus nidulans*, a non-sugarcane pathogenic fungus, indicating a close and specific interaction between *D. saccharalis* and *F. verticillioides* [21, 22].

This close association between an opportunistic fungus and the sugarcane borer led us to investigate the role of *D. saccharalis* as a dissemination vector for *F. verticillioides*, possibly corroborating the “host manipulation hypothesis”. We specifically addressed whether the fungus modifies insect behavior in ways that increase the likelihood of the fungus being disseminated by contaminated insects. Additionally, we evaluated whether this interaction benefits both organisms or only the fungus. Our results showed that *D. saccharalis* caterpillars are attracted to the VOCs emitted by *F. verticillioides* itself. Females not carrying the fungus prefer to lay their eggs on fungus-infected plants while females carrying the fungus prefer to lay their eggs on mock plants. In addition, *D. saccharalis* vertically transmits the fungus *F. verticillioides* to its offspring. In this scenario, our study indicates that *F. verticillioides* is not just an opportunistic fungus, but also a fungus more intimately associated with an insect herbivore, manipulating the plant and the insect to increase its potential for dissemination.

## Materials and methods

### Fungal culture and insect rearing

The fungus *F. verticillioides* was isolated from sugarcane plants and cultivated in potato dextrose (PD) medium (Difco, Sparks, NV, USA) at 25 °C with a 12 h photoperiod in climatic chambers. *A. nidulans* (A4 strain) was used as a control because it is not involved in red rot disease. It was cultivated in minimal medium (MM) [24] and maintained in climatic chambers at 37 °C in the dark.

The *D. saccharalis* was provided by Prof. Dr. José R. P. Parra from the University of São Paulo, Piracicaba. The caterpillars were fed an artificial diet [25] and maintained in a room under controlled conditions (temperature 25 ± 4 °C, relative humidity 60 ± 10% and 14 h of light). Adults were kept in cages covered with white paper sheets, where the eggs were deposited, collected and sanitized with 1% copper sulfate solution daily. Newly hatched caterpillars were transferred to the artificial diet [25].

### Olfactory preference assay

Five days before the experiment, a total of 10^5^ fungal conidia of *F. verticillioides* or *A. nidulans* were inoculated in a Falcon tube (15 mL) containing 7 mL of MM. The negative control was sterile MM. Tubes containing fungus-colonized medium and control medium were placed at opposite ends of the Petri dish (15 cm diameter) bottom, lined with moistened filter paper. A group of ten third-instar *D. saccharalis* caterpillars was released in the central region of the arena. The choice was quantified in the end of the experiment when the caterpillar remained in the Falcon tube to feed. The medium in the tubes represents a food source, once the caterpillars find it, they remain in the chosen tube. The Petri dishes were closed, sealed and kept in a dark room for 5 h at 25 °C; then, the number of caterpillars inside each tube was recorded. The assay was also performed using third-instar *Spodoptera frugiperda*, to detect specific attractiveness, and with fifth-instar *D. saccharalis*, to find changes in insect behavior during different immature stages.

To confirm insect attraction to fungal volatiles, VOCs collected from *F. verticillioides* were used to attract *D. saccharalis*. This assay was performed as described; however, only the control medium was added to the tubes. The hexane solvent was removed from the samples using nitrogen gas and the fungal VOCs were eluted in mineral oil. In addition to the control medium, each tube contained a piece of cotton loaded with either 50 µL of an aerated sample of *F. verticillioides* VOCs or solvent control (mineral oil). The dishes were placed in the dark for 7 h at 25 °C. All assays were repeated 10 times. Statistical analyses were performed using *t*-test (*p* **<** 0.05).

### Collection and quantification of fungal VOCs

VOCs emitted by *F. verticillioides* and *A. nidulans* in the MM were collected using an ARS Volatile Collection System (ARS, Gainesville, FL, USA). Six replicates of control (sterile medium) and fungus-colonized media were placed in fully enclosed glass chambers (21.5 cm length × 4 cm internal diameter) connected to the ARS by Teflon hoses. Clean and humidified air was injected into the chambers at 0.6 L/min. An adsorbent polymer column (30 mg; Hayesep-Q, 80–100 mesh, Alltech Associates, Deerfield, IL, USA) was coupled to the outlet of the glass chamber. After 8 h of VOCs collection, the polymer columns were eluted with 150 µL of distilled hexane (Sigma Aldrich, St. Louis, MO, USA), and the samples were stored in vials and kept in a freezer at −30 °C until analysis. VOCs quantification was performed using gas chromatography coupled to a flame ionization detector (GC-FID; GC2010 Shimadzu, Kyoto, Japan) equipped with a low polarity stationary phase column (30 m × 25 μm × 25 mm; HP-5, Agilent J&W, Santa Clara, CA, USA). Nonyl acetate (≥97% purity, Sigma Aldrich, St. Louis, MO, USA) was added (5 µL of a 10 ng/µL solution) in each sample as an internal standard. One microliter of each sample was injected at 250 °C in splitless mode with helium as the carrier gas (24 cm/s). The oven temperature was held at 40 °C for 5 min, raised to 150 °C at 5 °C/min, maintained for 1 min at 150 °C and then increased to 250 °C at 20 °C/min. The relative amount of each compound was determined based on the nonyl acetate peak area using GC Solution software (version 2.32.00). The effect of treatment on VOCs emission was analyzed by the nonparametric Kruskal–Wallis test followed by Bonferroni post hoc analysis (*p* **<** 0.05), because data did not pass the assumptions of an analysis of variance (ANOVA).

### Plants and *F. verticillioides* infection

One-eyed seed sets of sugarcane (*Saccharum* spp. cv. ‘SP80–3280’) provided from the field were grown in plastic cups (0.9 L) filled with coconut fiber and N:P:K (10:10:10). The plants were maintained in an insect-free greenhouse under natural light and temperature variations.

Fifty-day-old plants were artificially inoculated with 100 μL of an *F. verticillioides* suspension at a concentration of 1 × 10^5^ conidia/mL with the aid of a sterile syringe. Subsequently, the wound was covered with plastic film to prevent the entry of other pathogens. The plants were allowed to grow for more 10 days. Only plants exhibiting symptoms were used in bioassays and analyses. The same procedure was performed with a mock, noninoculated plant, except that the solution contained no conidia. The induction treatment (mock) did not alter the behavior of *D. saccharalis* compared with the healthy plant. One day prior to experiments, the plants were transferred to the laboratory and maintained under controlled conditions (temperature 26 ± 4 °C, relative humidity 60 ± 10%, and 12 h of light) with supplementary light (120 μmols).

### Oviposition bioassay

For the oviposition bioassays, we used noncontaminated or *F. verticillioides*-contaminated *D. saccharalis* adults to test their preference between mock and *F. verticillioides*-infected sugarcane. To obtain contaminated adults, third-instar caterpillars were removed from the stock rearing diet and were kept on a similar sterile diet but without nipagin or formaldehyde. Five days before this transfer, a total of 10^5^ fungal conidia was added to the diet to allow fungal colonization. The insects were kept in the fungus-colonized diet throughout their life cycle (contaminated insects). Noncontaminated insects were fed a sterile diet. The diet was changed every two days to prevent bacterial contamination.

The dual-choice oviposition preference test was performed in cages (100 × 70 × 50 cm) covered with voile fabric in a room with a controlled environment (temperature 26 ± 4 °C, relative humidity 60 ± 10% and 12 h of light). Three mated couples for each replicate, *n* = 10, were released inside the cage at the beginning of the scotophase (18:00 h), where they could freely choose between mock and *F. verticillioides*-infected plants, placed equidistantly overnight. Thereafter, the number of eggs laid in each plant was registered. Egg viability was evaluated by the number of neonates resulting from each treatment. The experimental data were analyzed using the *t*-test (*p* < 0.05).

### Collection and quantification of sugarcane VOCs

Mock and *F. verticillioides*-infected plants (*n* = 6) were enclosed individually in a glass chamber (50 cm width × 36 cm hight) and connected to the ARS Volatile Collection System (ARS, Gainesville, FL, USA). Briefly, clean humidified air was pushed at 0.3 L/min into the glass chamber connected to a column containing an adsorbent polymer (30 mg; Hayesep-Q, 80–100 mesh, Alltech Associates, Deerfield, IL, USA), which was connected to a vacuum pump pulling air for 12 h (from 18:00 to 06:00) at the same flow rate. Thereafter, the polymer column was eluted with 150 µL of distilled hexane, and the samples were stored in glass vials at −30 °C until analysis. VOCs quantification were performed using GC-FID (GC2010 Shimadzu, Kyoto, Japan) equipped with a non-polar stationary phase column (30 m × 25 μm × 25 mm; Rtx-1; RESTEK, Bellefonte, PA, USA) with helium as the carrier gas (24 cm/s). Nonyl acetate was added (10 µL of a 10 ng/µL solution) in each sample as an internal standard. The GC oven program, quantitative method, and statistical analysis were performed according to the same parameters used for fungal VOCs quantification.

### Chemical identification of VOCs from *F. verticillioides* and sugarcane

Chemical identification was done in gas chromatography coupled to a mass spectrometer (GC-MS; GCQP-2010 Ultra, Shimadzu Corp., Kyoto, Japan), equipped with a non-polar stationary phase column (30 m × 25 μm × 25 mm; Rtx-1MS; RESTEK, Bellefonte, PA, USA), and helium as carrier gas (41.1 cm/s). One microliter of fungal and plant extracts was injected at 250 °C in splitless mode using GC oven program for VOCs quantification described above. Quadrupole ion source and transfer line were kept at 250 °C for electron impact analysis at 70 eV (35–270 m/z). Fungal and plant VOCs were tentatively identified based on a comparison of mass spectra with the library database (NIST11) and Kovats retention indices. When available, authentic standards were used to confirm the identification of compounds, which were: benzaldehyde, 1-octen-3-ol, 3-hexenol-acetate, 2-octen-1-ol, and phenethyl alcohol, all from Sigma-Aldrich (Merck KGaA, St. Louis, Missouri, USA).

### Experimental design for quantification of *F. verticillioides* and *A. nidulans* in *D. saccharalis* and for microscopy

For quantification of *F. verticillioides* and *A. nidulans* in *D. saccharalis* tissues and for microscopy experiments, we used the mutants *Fv:DsRed* (as described in a different section) and *An:GFP:mRFP* [26], respectively. Previous laboratory tests showed that feeding on a mutant fungus-colonized or wild-type fungus-colonized diet did not affect *D. saccharalis* behavior. To quantify *F. verticillioides* and *A. nidulans* in *D. saccharalis*, fourth-instar caterpillars were removed from the rearing diet [25] and inoculated in the same diet, but sterile and lacked nipagin and formaldehyde. Five days before this transfer, a total of 10^5^ fungal conidia of *F. verticillioides* or *A. nidulans* were added to the diet to allow fungal colonization. Quantification of fungi in *D. saccharalis* tissues was performed in the following stages: fifth-instar caterpillars, female and male pupae, female and male adults, eggs, third- and fifth-instar offspring caterpillars. For insects in larval stages, the gut was separated from the body for quantification. For offspring quantification, first-instar caterpillars were added into tubes containing a sterile diet and were fed this diet until quantification. All experiments were performed with sterile material under a fume hood to avoid contamination. Three biological replicates for each *D. saccharalis* stage were used for quantification. Pupae and adults were sterilized for 2 min in a 1% sodium hypochlorite solution and then they were placed in distilled water for 1 min to remove stuck materials and product remains, before any analysis. To quantify the fungus in the eggs, we used 150 eggs in each replicate. The external surface of the eggs was sterilized using a cotton wool moistened with 1% copper sulfate solution, which effectiveness was tested (Supplementary Fig. S1). For microscopic analyses, we used five biological replicates of fifth-instar caterpillar’s gut, pupae and adult males and females, and ten biological replicas of eggs. The experiment was repeated twice. The experiments were statistically analyzed by the *t*-test (*p* < 0.05).

### Quantification of *F. verticillioides* and *A. nidulans* in *D. saccharalis* tissues

Quantification of *F. verticillioides* and *A. nidulans* in *D. saccharalis* tissues was performed by the standard curve method using a StepOne Real-Time PCR System (Applied Biosystems, Waltham, MA USA) and Maxima SYBR Green/ROX qPCR Master Mix (2X) (Fermentas, Waltham, MA, USA) [23]. *F. verticillioides* standard curve was prepared using the plasmid pCR2.1 (TA Cloning Kit, Invitrogen) containing the *F. verticillioides* ITS (rDNA internal transcribed spacer) fragment (primers: forward 5’ GATGAAGAACGCAGCGAAAT 3’ and reverse 5’ GAGGCTTGAGGGTTGAAATG 3’, annealing temperature 60 °C), as previously described [23]. *A. nidulans* standard curve was prepared using gDNA [27] and primers for tubulin gene (tubC) [28]. The standard curves consistently demonstrated correlation coefficients (*R*^2^) of 0.99 and PCR efficiencies over 90% when analyzed using StepOne software, version 2.0 (Applied Biosystems, Waltham, MA, USA).

### Insect and fungal DNA extraction

Insect DNA extraction was performed using the DNeasy Blood & Tissue Kit (Qiagen, Germantown, MD, USA) according to the manufacturer’s guidelines. Fungal DNA extraction was performed as previously described [29]. Total DNA was quantified using a NanoDrop 2000 (Thermo Scientific, Wilmington, DE, USA), and the quality was assessed by agarose gel electrophoresis.

### Identification of fungi in *D. saccharalis* by microscopy

For microscopic analyses, we used insects reared on the control, *Fv:DsRed*-colonized or *An:GFP:mRFP*-colonized diet. The offspring were reared only on the control diet. Using forceps and a scalpel, the gut of *D. saccharalis* third-instar caterpillars were removed and mounted on the slides using water. The pupae and eggs were analyzed in their integral form. For adults, the internal content was analyzed due a cut and removal of the abdomen region. The images were analyzed with an Olympus FV1000 confocal laser scanning microscope (Olympus, Center Valley, PA, USA) at room temperature using a 40× magnification objective lens. We used a filter for mRFP (excitation at 550/25 nm and emission at 605/70 nm). The images were analyzed using Olympus Fluoview FV1000-ASW software and saved as TIFF files.

### Fungal transformation

The *A. nidulans* (strain AGB655) expressing two fluorescent proteins (GFP/mRFP) was provided by Prof. Gustavo Henrique Goldman from the University of São Paulo, Ribeirão Preto. The strain was produced as previously described [26] and was used as a control in microscopy assays. The *A. nidulans* mutant (*An:GFP:mRFP*) was grown in MM supplemented with 2.5 µM pyridoxine.

*The F. verticillioides* was transformed via *Agrobacterium tumefaciens*-mediated transformation with DsRed using the vector pCAM-DsRed [30]. The *A. tumefaciens* strain EAH105 containing the plasmid pCAM-DsRed [30] was kindly provided by Prof. Dr. Maria Carolina Quecine Verdi from the University of São Paulo, Piracicaba. The transformation was performed as previously described [31], with few modifications. *A. tumefaciens* was added into 15 mL of liquid YEP medium with 100 µg/mL kanamycin and 100 µg/mL rifampicin and was incubated overnight at 28 °C with stirring at 200 rpm. Then, the cells of *A. tumefaciens* were diluted to an optical density (OD_660_) of 0.1 in 20 mL of inducing medium (IM) and then, 1 M 2-(N-morpholine) ethanesulfonic acid was added. The cultures were incubated at 28 ± 2 °C until an OD_660_ of 0.4 was obtained. Suspensions of *A. tumefaciens* (100 µL) and *F. verticillioides* (100 µL of a 10^6^ spores/mL suspension) were mixed on nitrocellulose membranes (BioRad, Berkeley, CA USA) in Petri dishes containing solid IM, 1 M 2-(N-morpholine) ethanesulfonic acid and 200 µL acetosyringone. The plates were incubated at 24 °C for 72 h. Then, the membranes were placed in BDA medium with hygromycin B (300 µg/mL) and sodium cefoxitin (200 µg/mL) for selection. Analysis of the transformants by fluorescence microscopy and evaluation of the transformants for mitotic stability were performed as previously described [31].

### Performance of *D. saccharalis* in *F. verticillioides*-infected plants

After 48 h of starvation, third-instar caterpillars were weighed and placed in individual cages attached to the base of sugarcane stems (one caterpillar per plant per treatment: mock and *F. verticillioides*-infected plants, *n* = 10). After 72 h, the caterpillars were carefully removed from the stem and weighed again. The assay results were analyzed using the *t*-test (*p* < 0.05).

### Performance of *D. saccharalis* in *F. verticillioides*-colonized diet

The *D. saccharalis* biological experiments were conducted using individuals fed on a *F. verticillioides*-colonized diet and control as described before, initially with 100 individuals each treatment. The experiment was kept under controlled conditions (temperature 26 ± 4 °C, relative humidity 60 ± 10% and 12 h of light). For each treatment, the following parameters were observed: egg phase, % hatching, total larval duration, larval viability, pupal stage duration, adult emergence percentage, sex ratio, total number of eggs per female, and longevity of males and females adults. The caterpillars were observed daily, and dead caterpillars were eliminated. After the transformation occurred, each pupa obtained was separated by sex. Males and females that emerged on the same day were placed in mating cages (1 couple/cage) as described in insect rearing. Mating combinations were formed (noncontaminated, both contaminated, female contaminated and male contaminated) in single-pair mating. A night light camera was used to record the observations. We evaluated the copulation latency, copulation duration, number of eggs, viability of eggs and survival of female and male adults. The life table parameters were analyzed according to a previously described method [32, 33]. In the single-pair mating experiments (*n* = 10), the parameters were analyzed by Tukey’s HSD test (*p* < 0.05), and survival was analyzed by the Kaplan–Meier method and compared using the log-rank test.

## Results

### Caterpillar attraction to fungal VOCs

**Fig. 1:**
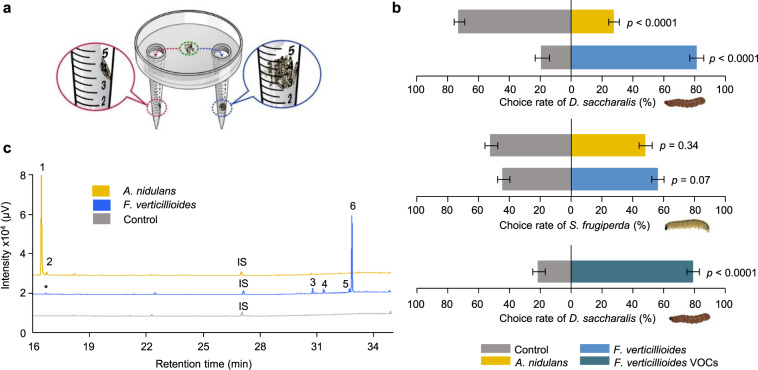
Insect attraction by fungal VOCs. **a** Design of olfactory dual-choice assay. This figure shows how the dual-choice olfactory preference assay was performed. We placed Falcon tubes (15 mL) with a sterile diet (control) or fungus-colonized diet on opposite sides of a Petri dish. The green circle indicates the caterpillar release area. The choice was quantified in the end of the experiment when the caterpillar remained in the Falcon tube to feed. **b** *Diatraea saccharalis* and *Spodoptera frugiperda* caterpillars, in a dual-choice assay with sterile diet on one side (control) and *Fusarium verticillioides*- or *Aspergillus nidulans*-colonized diet on the opposite side (upper and middle panels respectively) and *D. saccharalis* dual-choice assay using the *F. verticillioides* VOCs on one side and the solvent (control*)* on the opposite side (lower panel). Values are the means (±SEs) of ten replicates. Statistical analysis was performed using a *t*-test considering significance levels of *p* < 0.01. **c** Chromatographic profiles of volatiles released by *F. verticillioides, A. nidulans* and the sterile diet as a control. Internal standard (IS, nonyl acetate). (1) 1-Octen-3-ol, (2) unknown 1, (3) acoradiene, (4) unknown 2, (5) unknown 3, (6) acorenol. Asterisks designate the same compound present in both treatments.

VOCs play an essential role in plant-insect-fungus interactions and can influence insect behavior, acting as attractants or repellents [3]. To test the effect of fungal VOCs on *D. saccharalis* behavior, we performed a dual-choice olfactory preference assay (Fig. 1a) using third-instar caterpillars. The caterpillars preferred an *F. verticillioides*-colonized diet over a sterile diet (Fig. 1b; and Supplementary Video 1). The *A. nidulans* fungus is not related to sugarcane diseases, and caterpillars challenged by *A. nidulans* VOCs preferred the control over the fungus-colonized diet, indicating either no preference or a repellent effect (Fig. 1b). The same type of assay was performed with *D. saccharalis* fifth-instar caterpillars, which were also attracted to *F. verticillioides* VOCs (Supplementary Fig. S2), indicating that the attraction was consistent across different larval stages. A similar dual-choice olfactory preference assay was performed using *Spodoptera frugiperda* caterpillars to evaluate the specificity of the VOCs. In this assay, *S. frugiperda* caterpillars were not attracted to *F. verticillioides* or *A. nidulans* VOCs (Fig. 1b), supporting our hypothesis of a close and specific interaction between *D. saccharalis* caterpillars and *F. verticillioides*.

VOCs collected from *F. verticillioides* grown in vitro were offered as attractants to *D. saccharalis* caterpillars. More than 80% of the *D. saccharalis* caterpillars preferred the *F. verticillioides* VOCs (Fig. 1b), supporting our previous assays and suggesting a role for *F. verticillioides* VOCs in attracting *D. saccharalis*.

VOCs emitted by fungi grown in vitro were analyzed by GC-MS and quantified by GC-FID. Acorenol, acoradiene, two unknown compounds (unknown 2 and 3) and a small amount of 1-octen-3-ol were identified in *F. verticillioides* VOCs. Only two compounds were identified in *A. nidulans* VOCs: over 90% was 1-octen-3-ol, and the remaining part was an unknown compound (unknown 1) (Fig. 1c and Supplementary Table S1). Although 1-octen-3-ol was present in both blends of VOCs, differences in the concentration and proportion of blend components may explain the opposite effects on caterpillar behavior [34] (Supplementary Fig. S3).

### Insect and plant modulation by the fungus

**Fig. 2:**
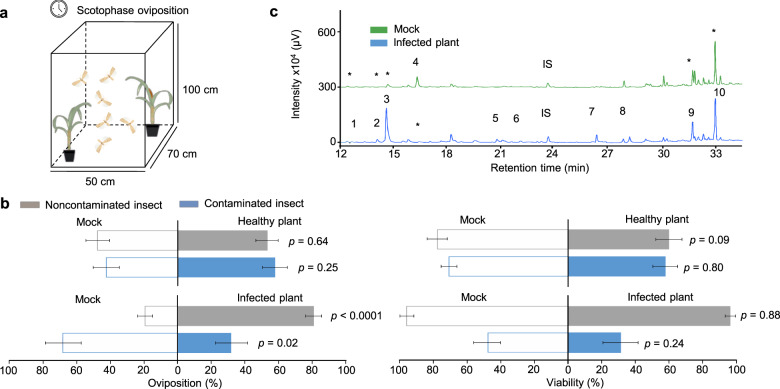
Performance of *Diatraea saccharalis*. **a** Cages assays in which the moths were simultaneously presented with mock versus *Fusarium verticillioides*-infected plants. **b** Noncontaminated insects (fed a sterile diet) and contaminated insects (fed a fungus-colonized diet) choice for oviposition and egg viability from mock (manipulated plant) and healthy plants; mock and *F. verticillioides*-infected plants. Statistical analysis was performed using a *t*-test considering significance levels of *p* < 0.05. Values are the means (±SEs) of 10 replicates. **c** Chromatographic profiles of VOCs released by mock and *F. verticillioides*-infected plants. Internal standard (IS, nonyl acetate). (1) Benzaldehyde, (2) 3,5,5-trimethyl-1-hexene, (3) 1-octen-3-ol, (4) 3-hexenyl-acetate, (5) 2-octen-1-ol, (6) phenethyl alcohol, (7) unknown 4, (8) acorenol, (9) 6.10.14-trimethyl-2-pentadecanone, (10) unknown 5. Asterisks designate the same compound in both treatments.

To check the influence of *F. verticillioides*-infected plants on *D. saccharalis* behavior, oviposition preference of female adults was assessed in a dual-choice assay (Fig. 2a). Interestingly, when females were previously fed on a diet without fungus, they preferred *F. verticillioides*-infected plants for oviposition (80.84%) over mock plants (19.15%) (*t* = 78.29, *p* < 0.0001) (Fig. 2b). Females previously fed on *F. verticillioides*-colonized diet preferred mock plants for oviposition (68.23%) over *F. verticillioides*-infected plants (31.77%) (*t* = 5.99, *p* = 0.02) (Fig. 2b). Egg viability was unaltered by *F. verticillioides* infection (*t* = 0.02, *p* = 0.88; *t* = 1.52, *p* = 0.24) (Fig. 2b). These results indicate that infection by *F. verticillioides* directly modifies the host preference of moths in ways that support fungal dissemination.

Given the vital role of VOCs in the host preference of moths, we evaluated the changes in VOCs emissions in *F. verticillioides*-infected plants. Compared to the mock treatment, the VOCs profile of *F. verticillioides*-infected plants changed not only quantitatively but also qualitatively (Fig. 2c and Supplementary Table S2). Among the 10 compounds found in the sugarcane VOCs profile, 2-octen-1-ol, phenethyl alcohol, acorenol and an unknown compound (unknown 4) were present in only *F. verticillioides*-infected plants. These plants also released higher concentrations of 3,5,5-trimethyl-1-hexene and 1-octen-3-ol than the mock plants (Bonferroni, *p* < 0.05). Acorenol and 1-octen-3-ol seem to be released by *F. verticillioides* itself, indicating a possible role of these compounds in insect behavior.

The antennae of *D. saccharalis* moth were challenged against synthetic VOCs identified in this study, when it was commercially available. The electroantennography (EAG) response showed that all tested compounds increased the effect of dose-response: 1-octen-3-ol (χ^2^ = 22.82, *p* < 0.001), 3-hexenyl-acetate (χ^2^ = 36.49, *p* < 0.001), 2-octen-1-ol (χ^2^ = 35.29, *p* < 0.001), benzaldehyde (χ^2^ = 51.52, *p* < 0.001) and phenethyl alcohol (χ^2^ = 33.36, *p* < 0.001) (Supplementary Fig. S4) which confirm the same background response for all treatments. However, only for 1-octen-3-ol compound, the EAG signal was significantly reduced in noncontaminated females when compared to contaminated females (χ^2^ = 9.22, *p* = 0.002), indicating a greater sensitivity to this compound when the insects are contaminated by *F. verticillioides* (Supplementary Fig. S4).

### Vertical transmission of *F. verticillioides*

**Fig. 3:**
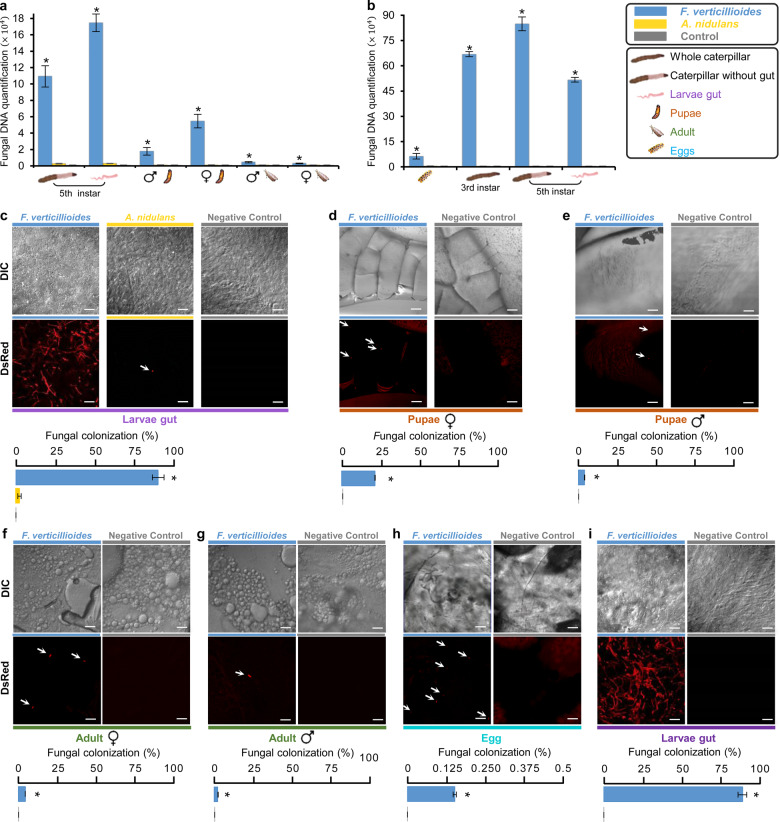
Evidence of vertical transmission of *Fusarium verticillioides* by *Diatraea saccharalis*. **a** Quantification of *F. verticillioides* (ITS, rDNA internal transcribed spacer) and *A. nidulans* (TubC gene) in different life cycle stages of *D. saccharalis* previously fed on a sterile (control), *F. verticillioides*-colonized or *A. nidulans*-colonized diet. **b** Quantification of *F. verticillioides* (ITS) in *D. saccharalis* offspring grown on a sterile diet. Values for **a** and **b** are the means (±SEs) of three biological replicates. Asterisks represent significant differences (*t*-test, *p* < 0.05) in comparison with the control. **c–g** Identification of *F. verticillioides* (*Fv:DsRed*) and *A. nidulans* (*An:GFP:mRPF*) tagged with the red fluorescent protein in different stages of *D. saccharalis* development after feeding on the sterile (control), *F. verticillioides*-colonized or *A. nidulans*-colonized diet. **h** and **i** Identification of *Fv:DsRed* in *D. saccharalis* offspring by microscopy. The offspring caterpillars were fed on a sterile diet. For **c** to **i**, the bars represent 50 µm, and the graphs represent *D. saccharalis* area showing fungal colonization. Values are the means (±SEs) of five biological replicates, except for eggs that were used ten replicates. Asterisks represent significant differences (*t*-test, *p* < 0.05) in comparison with the control. Arrows indicate the presence of *Fv:DsRed* spores.

To assess a potential dissemination benefit to the fungus, vertical transmission through *D. saccharalis* was investigated. Our data showed that *F. verticillioides* DNA acquired by caterpillars during feeding on a fungus-colonized diet was present throughout the caterpillar life cycle (Fig. 3a). Quantification of *F. verticillioides* DNA showed an increased level of fungi in caterpillars and pupae, with reduced levels in adults. *F. verticillioides* DNA was also detected in *D. saccharalis* offspring (Fig. 3b). The amount of *F. verticillioides* DNA in *D. saccharalis* eggs was 5.9×10^4^ times greater than that in the control. The offspring caterpillars presented higher levels of fungal contamination than the eggs (Fig. 3b). The high levels of fungal DNA in the offspring caterpillars could be explained by the consumption of a diet colonized by fungi by the newly hatched caterpillars. Although a sterile diet was used for rearing *D. saccharalis* offspring, the caterpillars released the fungus into the diet, which was confirmed by microscopy (Supplementary Fig. S5), and then consumed the fungus again. *A. nidulans* was also present in *D. saccharalis*, but only in the larval stage, when the caterpillar was in direct contact with the fungus, and this fungus was not vertically transmitted, in contrast to *F. verticillioides* (Fig. 3a, b).

When caterpillars were fed on a diet colonized by *F. verticillioides* tagged with red fluorescent protein (*Fv:DsRed*), the fungus was detected in approximately 90% of the *D. saccharalis* gut (Fig. 3c). Caterpillars fed on a diet colonized by *A. nidulans* tagged with red fluorescent protein (*An:GFP:mRPF*) showed the fungus in only 2.5% of the gut area (Fig. 3c). *Fv:DsRed* was detected in 21.4% of the internal material of female pupae, and in 4.1% of male pupae (Fig. 3d, e, Supplementary Fig. S6). In adults, the presence of 5.1% of *Fv:DsRed* was identified inside the abdomen of females and 2.3% in males (Fig. 3f, g). The presence of *Fv:DsRed* on the egg surface was not detected by microscopy (Supplementary Fig. S7), but it was detected internally in the eggs (Fig. 3h and Supplementary Fig. S8). In the offspring caterpillars the fungus was detected in approximately 90% of the intestine area, the same value identified in this stage for the genitors (Fig. 3i). These data were supported by qRT-PCR quantification, showing vertical transmission of *F. verticillioides* to the offspring.

The ability of *D. saccharalis* to transmit the fungus *F. verticillioides* to the plant was analyzed using the offspring from genitors reared on an *Fv:DsRed*-colonized diet. The offspring were reared on sterile diet and had no contact with fungal colonization. When the contaminated offspring (from genitors reared on an *Fv:DsRed*-colonized diet) attacked the plant, they were able to transmit the fungus (*Fv:DsRed*), which was isolated from the plant and visualized by microscopy (Supplementary Fig. S9). Sixty percent of the plants inoculated with these caterpillars showed fungal infection after a period of 12–15 days. The plants attacked by noncontaminated offspring (from genitors reared on a sterile diet) showed no fungal dissemination, suggesting that vertical transmission of the fungus associated with vector activity plays an important role in the epidemiology of *F. verticillioides* in sugarcane.

### Influence of the fungus on insect performance

**Fig. 4:**
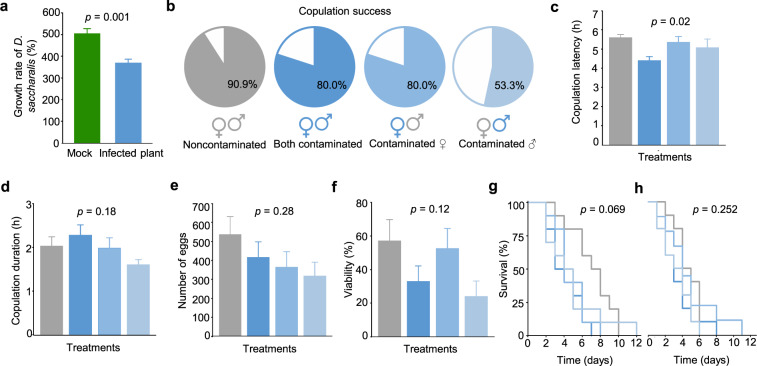
Influence of the fungus on *Diatraea saccharalis* performance. **a** Effect of *Fusarium verticillioides* on *D. saccharalis* performance in plants. Values are means of the growth rate (±SEs, *n* = 10). Statistical analysis was performed using a *t*-test considering significance levels of *p* < 0.05. From **b** to **h**, Performance of *D. saccharalis* fed on *F. verticillioides*-colonized diet or the control, sterile diet. **b** Copulation success of single pairs of *D. saccharalis* following all combinations of treatments, noncontaminated, both contaminated, contaminated female, contaminated male. **c** Copulation latency. **d** Copulation duration. **e** Number of eggs. **f** Egg viability. Survival of female **g** and male **h** adults. Values are the means (±SEs, *n* = 10). Statistical analysis was performed using a Tukey’s test considering significance levels of *p* < 0.05. Survival curves were calculated by the Kaplan–Meier method and compared by the log-rank test.

To assess whether developing *D. saccharalis* insects incur fitness costs by carrying *F. verticillioides*, the herbivore’s performance was measured in terms of larval growth rate while feeding on infected sugarcane plants. After feeding for four days, third-instar *D. saccharalis* caterpillars gained less weight when fed on *F. verticillioides*-infected plants than those fed on mock plants (*t* = 8.10, *p* = 0.001; Fig. 4a). We tested whether this would also result in fitness costs for *D. saccharalis*, a scenario that would interfere with fungal dissemination. Indeed, when we kept the caterpillars on the *F. verticillioides*-colonized diet, the mean net reproductive rate, i.e., the ability to generate females (R_0_), was significantly lower (66.19) than that of caterpillars fed the control, sterile diet (119.02) (Supplementary Table S3). However, the mean generation time (T) (*t* = 0.65, *p* = 0.42) was not affected by feeding with an *F. verticillioides*-colonized diet. The same result was observed for the intrinsic rate of increase (r_m_) and the finite rate of increase (λ). Although we did not observe major effects on moth biological parameters, we assessed the potential effects of *F. verticillioides* on *D. saccharalis* mating behavior. To this end, we used the single-pair mating assay in which it was possible to evaluate all combinations of male and female adults, contaminated or not by the fungus. Interestingly, contamination resulted in decreased copulation success, irrespective of whether the female, the male, or both moths were contaminated (Fig. 4b). In addition, contamination appeared to alter the mating time of the moths. The mating latency period, when both males and females were contaminated, was shorter by at least 1 h compared to that for the combinations in which one of the adults was not contaminated (*F* = 3.74, *p* = 0.02) (Fig. 4c). The duration of copulation (*F* = 1.71, *p* = 0.18), number of eggs (*F* = 1.31, *p* = 0.28), viability of eggs (*F* = 2.01, *p* = 0.12), survival of females (χ^2^ = 7.09, *p* = 0.06) and survival of males (χ^2^ = 4.09, *p* = 0.25) were not affected by fungal contamination (Fig. 4d–h).

## Discussion

Here, we present evidence of how a fungus gains dissemination benefits by modulating its host’s VOCs and herbivorous insect responses to these VOCs. The phytopathogenic fungus *F. verticillioides* releases VOCs that are attractive to *D. saccharalis* caterpillars; furthermore, the fungus modulates the emission of plant volatile compounds by changing the VOCs profile of infected plants. The EAG results suggest a possible mechanism occurring at the level of the peripheral nervous system, modulating the insect behavior in a way that only noncontaminated insects seek *F. verticillioides*-infected plants. The confirmed vertical transmission of the fungus by contaminated *D. saccharalis* adults and eggs assists fungal dissemination to other plants representing an essential benefit for the fungus. Altogether, our data show that these behaviors are conducive to fungal inoculation into the plant as well as mechanical transmission, being consistent with the “host manipulation hypothesis” [4].

Several studies have shown interactions between fungi and insects in plant colonization [35–40]; however, fungus-vector insect interactions remain poorly understood. Microorganisms can be carried by insects in their gut, exoskeleton, hemocoel, malpighian tubules, peripheral tissues, abdominal region, and cytoplasm and even in the cell nucleus [41–44], and they can be vertically transmitted in capsules [45], secretions [46] and excretions [47–50]. *F. verticillioides* is ingested by caterpillars, passing through their digestive tract and being transferred to their offspring, increasing the potential for infection of healthy plants. However, the mechanism by which the fungus remains in insect tissues remains unknown.

The changes in plant VOCs emission after fungal infection associated to the change in insect preference after fungus acquisition showed in this work was previously studied in interactions with vector-borne viruses, such as barley yellow dwarf virus (BYDV) [51], tomato spotted wilt virus (TSWV) [9], and potato leaf roll virus (PLRV) [52, 53], as well as bacteria [54]. However, in this work we showed a phytopathogenic fungus that uses the strategies previously demonstrated by vector-borne virus and bacteria [9, 51–54] to manipulate both plant and vector interactions and being vertically transmitted to the vector’s offspring.

The vertical transmission of fungi in insects is not well understood, while a great majority of reports related to symbiont transmission mechanisms are restricted to bacteria and archaea [18]. The interaction of *D. saccharalis* and *F. verticillioides* apparently starts with the release of fungal VOCs which are recognized by *D. saccharalis*, guiding larvae and adults toward fungus-infected plants, where tactile and taste clues can also take a place. The first contact of the sugarcane borer with the fungus occurs when the caterpillars feed on fungus-infected plants, suggesting horizontal transmission of the fungus; however, the presence of the fungus in the gut, adults and eggs demonstrates that after ingestion by the caterpillars, the fungus is vertically transmitted to the offspring.

The plant-insect-fungus interaction studied here is beneficial to fungus dissemination, but in contrast to other interactions, in which pathogen infection provides superior nutritional quality for the vector’s offspring [55], *F. verticillioides* infection in sugarcane does not directly benefit the insect. Insects fed on fungus-infected plants showed a poorer performance than those fed on mock plants. The observed lower weight gain might be related to antinutritional plant defense responses in this multitrophic interaction. Despite the weight loss, *D. saccharalis* completed its life cycle, thus providing a full advantage to the fungus. Moreover, sugarcane plants infected by *F. verticillioides* release less attractive herbivore-induced plant VOCs to *Cotesia flavipes*, a parasitoid of the *D. saccharalis* caterpillar [56], indicating an indirect benefit to the herbivore of co-occurring with the fungus. The strategy of feeding in a safe environment, provided by another species was also studied with brown planthopper. This insect takes advantage of the VOCs released by plants previously attacked by the rice striped stem borer, which are avoided by the egg parasitoid *Anagrus nilaparvatae*, an enemy of brown planthopper [57]. Therefore, for *D. saccharalis*, the cost of feeding in *F. verticillioides*-infected plants can be compensated by avoiding *C. flavipes* parasitoid. These observations highlight the influence of *F. verticillioides* on plant and insect modulation.

Previous studies have shown that other *Fusarium* species can also produce a series of VOCs, such as alcohols, esters, and aldehydes, which are either attractive or repellent to different insect species [38, 58, 59]. Nevertheless, as demonstrated in this study, VOCs-elicited attraction is only the first step of a multifaceted interaction. Our results support a paradigm change in which the fungus is not merely an opportunistic player, but also manipulates both the host plant and the insect throughout its life cycle to promote infection and dissemination.

## Supplementary information

Supplementary video

Supplementary information
