# Supplementary material for: Fungal phytopathogen modulates plant and insect responses to promote its dissemination
Source: ISME J. 2021 Jun 14;15(12):3522–33. doi: 10.1038/s41396-021-01010-z (PMC8630062; doi:10.1038/s41396-021-01010-z)
Supplement: Supplementary file 2 — Supplementary information [file 41396_2021_1010_MOESM2_ESM.docx]

**Supplementary Information**

Fungal phytopathogen modulates plant and insect responses to promote its dissemination

Flávia P. Franco^1*^, Amanda C. Túler^2*^, Diego Z. Gallan^1*^, Felipe G. Gonçalves^2^, Arodí P. Favaris^2^, Maria Fernanda G. V. Peñaflor^3^, Walter S. Leal^4^, Daniel S. Moura^5^, José Maurício S. Bento^2^, Marcio C. Silva-Filho^1^

^1^Departamento de Genética, Escola Superior de Agricultura Luiz de Queiroz, Universidade de São Paulo, Av. Pádua Dias, 11, 13180-900 Piracicaba, SP, Brazil.

^2^Departamento de Entomologia e Acarologia, Escola Superior de Agricultura Luiz de Queiroz, Universidade de São Paulo, Av. Pádua Dias, 11, 13418-900 Piracicaba, SP, Brazil.

^3^Departamento de Entomologia, Universidade Federal de Lavras, Av. Dr. Sylvio Menicucci, 1001, 37200-000 Lavras, MG, Brazil.

^4^Department of Molecular and Cellular Biology, University of California, One Shields Avenue, 95616 Davis, CA, USA.

^5^Departamento de Ciências Biológicas, Escola Superior de Agricultura Luiz de Queiroz, Universidade de São Paulo, Av. Pádua Dias, 11, 13418-900 Piracicaba, SP, Brazil.

May 4, 2021

This file includes:

Materials and methods

Supplementary Figures S1-S9

Supplementary Tables S1-S3

Supplementary Video 1 Legend

_______________________

^*^These authors contributed equally.

**Materials and methods**

**Efficiency of egg surface sterilization by copper sulfate (CuSO_4_).**

To evaluate the effectiveness of the copper sulfate solution in the *Diatraea saccharalis* egg surface sterilization, we analyzed the fungal growth on Petri dishes containing PDA medium (Difco, Sparks, NV, USA) with penicillin (100 µg/mL), tetracycline (50 µg/mL) and 1% copper sulfate (negative control) and without sulfate copper (positive control). For this experiment, *Fusarium verticillioides* mycelium was inoculated into Petri dishes containing PDA medium and, after growth, squares (25 mm²) were removed and inoculated in negative control plates and positive control plates. Eggs from *D. saccharalis* and macerated content of *D. saccharalis* eggs (the posture was macerated with the aid of a glass pistil, afterward, 250 µL of Milli-Q water was added followed by 30 s vortex, the entire content was inoculated in 5 points of the plate) were used to verify the efficiency of egg surface sterilization. The plates were kept at 24°C with a photoperiod of 12 hours of light for 4 days. Three replicates were used, the experiment was repeated twice. The results showed that sterilization with 1% copper sulfate is efficient to eliminate *F. verticillioides* from *D. saccharalis* eggs. 100% of the plates containing copper sulphate (1%) did not show fungal growth, while positive control plates showed fungal growth (Supplementary Fig. S1).

Electroantennogram

For the sensitivity test detection, we used an EAG system induced by the stimuli or control puffs recorded in software EAGPro version 2.0 (Syntech, Germany). Preparation was bathed in a high humidity air stream flowing at 30 mL/s to which a stimulus pulse of 1 mL/s was directed into antennae. The antenna of female *D. saccharalis* mated was fixed between two stainless steel electrodes using conductive gel (Signa gel, Parker Labs., USA), and the last articles of the distal portion of the antenna were cut to facilitate electrical contact. All compounds (benzaldehyde, 1-octen-3-ol, 3-hexenol-acetate, 2-octen-1-ol, and phenethyl alcohol) were dissolved in paraffin oil in concentrations 20, 200, and 2.000 ppm. An aliquot (10 μL) of each compound and concentration was loaded onto a filter paper strip (2×6 mm), which was immediately inserted into a Pasteur pipette and delivered to the antennae. Solvent (paraffin oil) alone was used as control. For each compound, the EAG responses of six female moths were recorded. Statistical analyzes were performed using the generalized mixed linear models, with *Gamma* distribution, the fixed variable being the treatments (noncontaminated and contaminated insects) and the random variable the concentration of the evaluated compound (Supplementary Fig. S4).

**Evidence of vertical transmission of *Fusarium verticillioides* by *Diatraea saccharalis* using fungal isolation and identification by laser confocal microscopy**

To evaluate the vertical transmission of *F. verticillioides* by *D. saccharalis*, we inoculated whole or macerated eggs of *D. saccharalis* in PDA medium, using the mutant *Fv:DsRed*. For this, the caterpillars of *D. saccharalis* were fed on a diet colonized by *Fv:DsRed,* as described in “Experimental design for quantification of *F. verticillioides* and *A. nidulans* in *D. saccharalis* and for microscopy”. The control consists of insects fed on a sterile diet. Approximately 25 eggs were collected after 7 days of oviposition. The eggs were individually packed in 2 mL microtubes and stored at -80 ºC. Afterwards, the postures were individually analyzed in the stereomicroscope with a 40X magnification, and the intact postures (absence of eggs with any sign of damage) were selected. They were inoculated in their whole form or macerated (as previously described) in plates containing PDA medium, penicillin (100 µg/mL) and tetracycline (50 µg/mL). The plates were kept at 24 °C and photoperiod of 12 hours of light for 4 days. After this period, the entire circumference of the inoculation site was sampled and the samples were transferred to glass slides containing ddH_2_O, which were covered with a coverslip. The slides were visualized in an Olympus FV1000 confocal microscope, using the mRFP filter to check the expression of the red fluorescence of DsRed gene, present in *F. verticillioides*, thus confirming its presence. The images were analyzed using the Olympus Fluoview FV10-ASW program. The 40X objective was used in all captured images. Three replicates were used, the experiment was repeated twice. Macerated *D. saccharalis* eggs from caterpillars fed on *F. verticillioides* contaminated-diet showed 100 % of fungal growth. The fungus was identified as *F. verticillioides* through the presence of red fluorescence detected by confocal microscopy. The negative control showed no fungal growth. The treatments with whole eggs did not show fungal growth for eggs from caterpillars fed on *F. verticillioides* contaminated-diet, and neither for eggs from caterpillars fed on the sterile diet. This result confirms the vertical transmission of *F. verticillioides* by *D. saccharalis* (Supplementary Fig. S8), and excludes the hypothesis of fungal transmission by egg surface. The experiment did not present any contamination with other microorganisms.

***Fusarium verticillioides* transmission to plants**

To determine whether *F. verticillioides* infection in sugarcane plants can be mediated by *D. saccharalis*, we used an *F. verticillioides* mutant (*Fv:DsRed*). For this, *D. saccharalis* genitors were fed an *Fv:DsRed*-colonized diet as described in the previous assay (Experimental design for quantification of *F. verticillioides* and *A. nidulans* in *D. saccharalis* and for microscopy) . The control consisted of genitors fed a diet lacking *Fv:DsRed*. The offspring from each treatment were reared on a sterile diet. However, the diet was changed daily to avoid new contamination. The offspring had no contact with fungal colonization. When the caterpillars reached the third-instar, they were removed from the rearing diet and placed in individual cages attached to the base of sugarcane stalks (one caterpillar per plant, n = 10) for 72 h. Samples were collected from 2 to 5 cm around the inoculation area 12-15 days after the introduction of the caterpillar. The sample surface was sterilized with alcohol (70%) for 30 s, followed by hypochlorite (0.5%) for 1.5 min and alcohol (70%) for another 30 s and then by 3 washes with sterile water. The samples were then plated on PD medium with hygromycin B (300 μg/mL) and sodium cefoxitin (300 μg/mL) and were incubated (26 °C, 60 ± 10% relative humidity and 12 h photoperiod) in climate-controlled chambers for 7 days. The fungal isolates were mounted in the water on slides, and fluorescence emission by *Fv:DsRed* was verified. We analyzed the images using an Olympus FV1000 confocal laser scanning microscope (Supplementary Fig. S9).

**Supplementary Figures S1-S9**

**Supplementary Fig. S1 Efficiency of egg surface sterilization by copper sulfate.** Inoculation of *Fusarium verticillioides* (**a**) or macerated *Diatraea saccharalis* eggs (**b**) in potato dextrose (PD) medium (positive control) or PD medium with 1% CuSO_4_ (negative control).


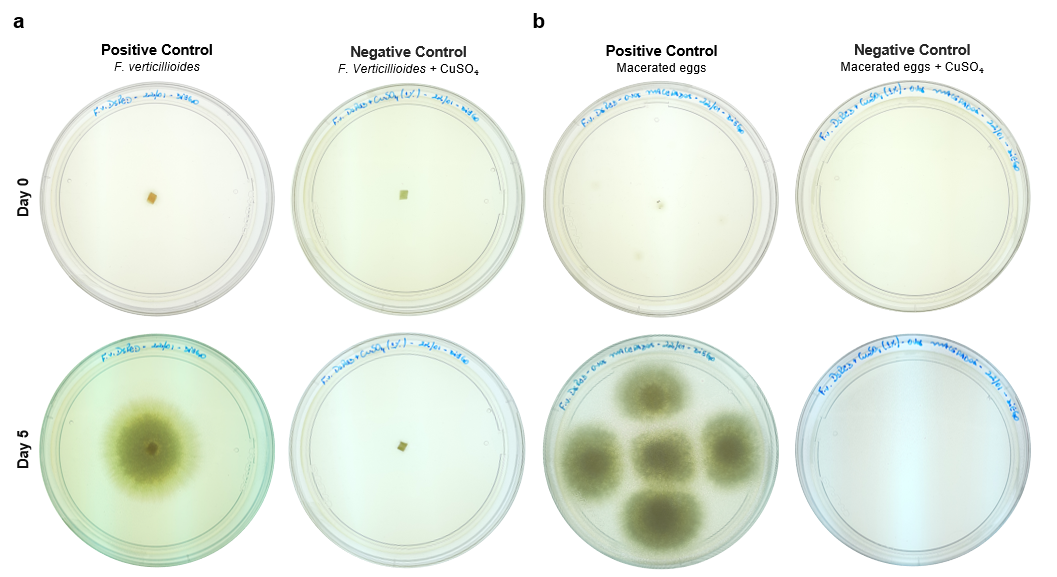


**Supplementary Fig. S2** **Olfactory choice assay with fifth-instar *Diatraea saccharalis* caterpillars.** Dual-choice assay comparing the sterile diet (control) and the *Fusarium verticillioides-* or *Aspergillus nidulans*-colonized diet. Asterisks represent significant differences (*t*-test, *p* < 0.05) in comparison with the control. Values are the means (± SEs) of ten replicates.


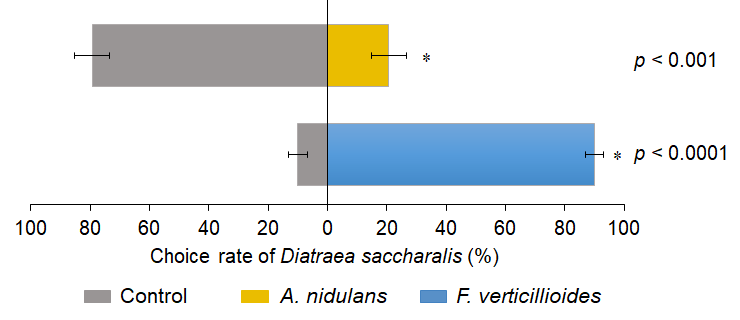


**Supplementary Fig. S3 Olfactory choice assay with third-instar *Diatraea saccharalis* caterpillars and 1-octen-3-ol.** *D. saccharalis* caterpillars, in a dual-choice assay, using the synthetic VOC 1-octen-3-ol in different concentrations on one side and the mineral oil solvent (control) on the opposite side. We used 10 µL of 1-octen-3-ol or mineral oil. Values are the means (± SEs) of ten replicates. Statistical analysis was performed using a *t*-test considering significance levels of *p* < 0.05. NS means statistically not significant.


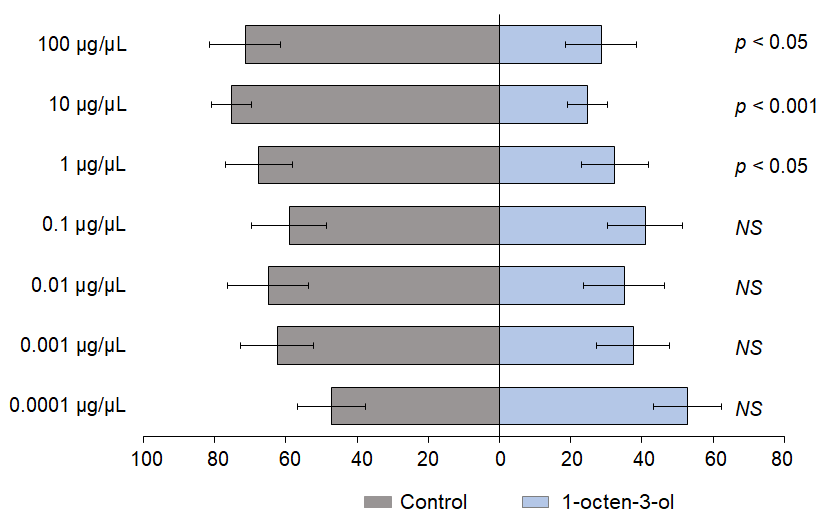


**Supplementary Fig. S4** **Electroantennogram (EAG) responses for plant VOCs in** ***Diatraea saccharalis* female moth*.*** **a** Schematic drawing of electroantennogram. The antenna was excised and set up in the EAG device. The puff containing the odorants was delivered to the antenna. The stimuli puffs were recorded by Syntech software (monitor screen). Traces showing EAG responses of (**b**) 1-octen-3-ol, (**c**) 2-octen-1-ol, (**d**) 3-hexenyl-acetate, (**e**) benzaldehyde and (**f**) phenethyl alcohol. The median is marked by a curve line. Non-contaminated means insect feed in a sterile diet*,* and contaminated means insect feed on *F. verticillioides*-colonized diet.


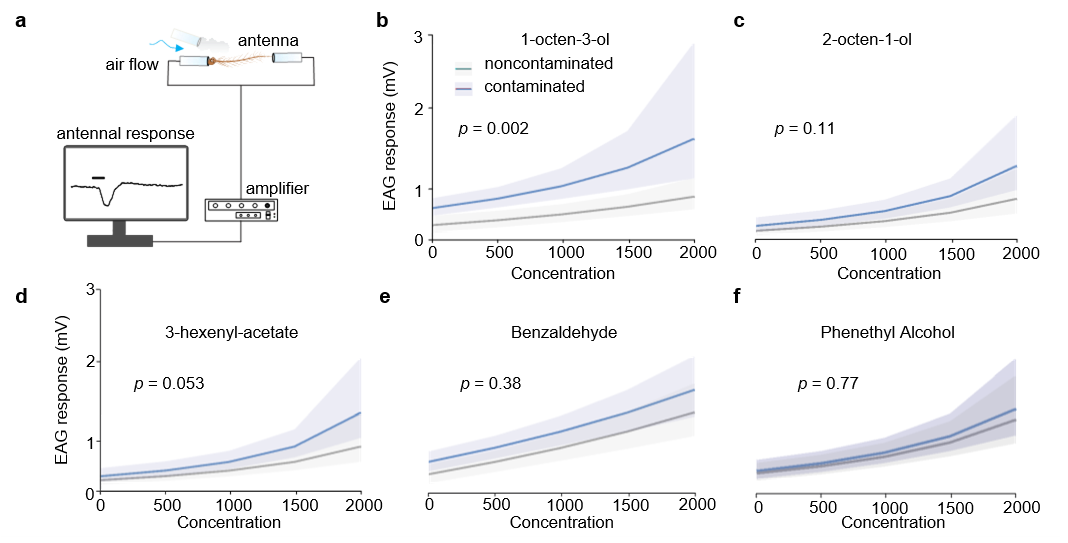


**Supplementary Fig. S5 *Fusarium verticillioides* dissemination in the diet by *Diatraea saccharalis* caterpillar.** **a** Confocal microscopy image of growing fungus (*Fv:DsRed*) in sterile diet after newly hatched caterpillar started feeding. The fungus was released by newly hatched caterpillars from genitors previously fed on *Fv:DsRed*-contaminated diet. Positive control, *Fv:DsRed* cultivated on PD media. Negative control, *F. verticillioides* wild type. Bars represent 50 μm. **b** Agarose gel electrophoresis with hygromycin (*hph*) gene (600 bp^55^) to confirm *Fv:DsRed* released by newly hatched caterpillars from genitors previously fed on *Fv:DsRed*-contaminated diet. Positive control, *E. coli* containing the hygromycin (*hph*) gene. Negative control, *F. verticillioides* wild type.

**Fungus release by caterpillars**


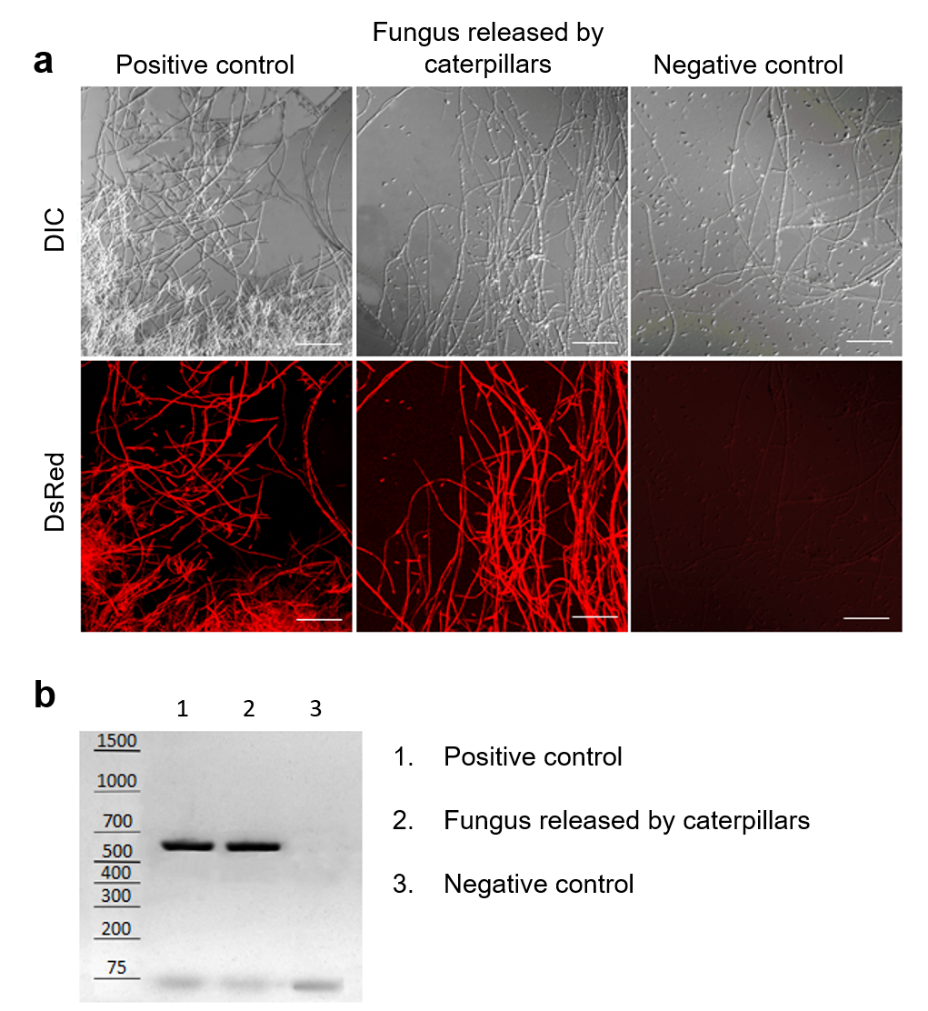


**1 2 3**

**1.** Positive Control

**2.** Fungus released by caterpillars

**3.** Negative Control

**DIC**

**DsRed**

**b**

**a**

**Negative Control**

**Positive Control**

**Supplementary Fig. S6 Identification of *Fusarium verticillioides* in *Diatraea saccharalis* pupae.** Identification of *F. verticillioides* tagged with the red fluorescent protein (*Fv:DsRed*), by confocal microscopy, in the internal content of a female (**a**) and male (**b**) pupae of *D. saccharalis*, from caterpillars fed on a sterile diet (control) or *F. verticillioides*-colonized diet. The pupa's internal content was removed using a syringe and visualized by confocal microscopy. The bars represent 50 µm. The arrows indicate the presence of *F. verticillioides* spores.

**
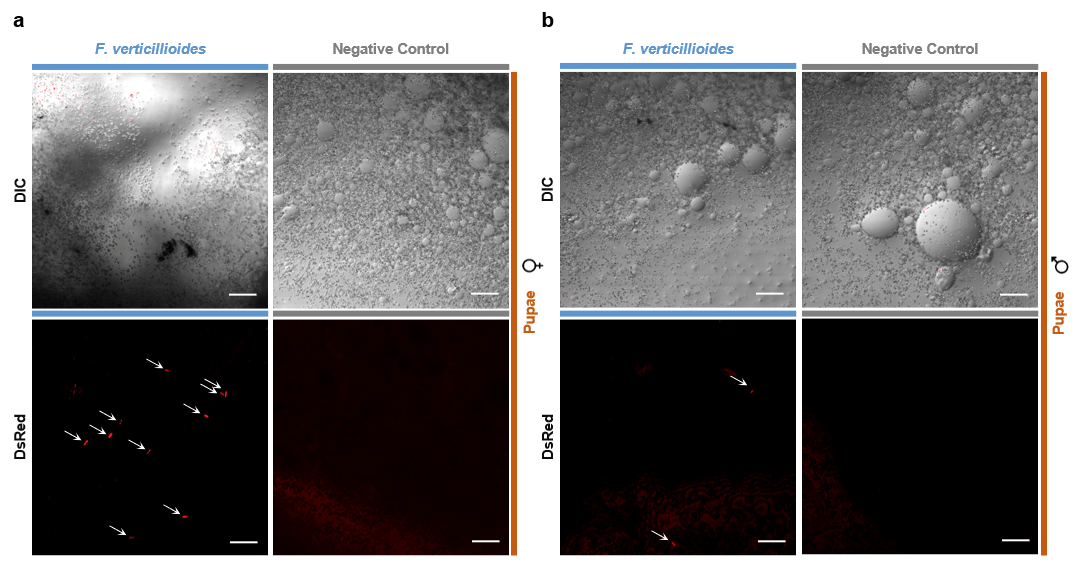
**

**Supplementary Fig. S7 *Diatraea saccharalis* eggs surface**. The surface of *D. saccharalis* eggs was visualized under confocal microscopy for detection of *Fusarium verticillioides*. Eggs from genitors previously fed on *Fv:DsRed*-contaminated diets (*F. verticillioides*) and from genitors previously fed on the sterile diet (negative control) were inspected. Bars represent 100 μm.

***F. verticillioides***

**Negative Control**


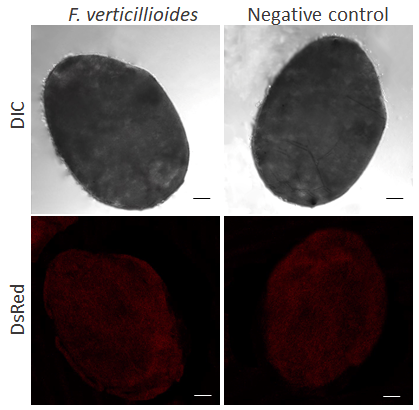


**DsRed**

**DIC**

**Supplementary Fig. S8 Vertical transmission of *Fusarium verticillioides* by *Diatraea saccharalis*. a** Inoculation of whole eggs of *D. saccharalis* (eggs from genitors fed on *F. verticillioides* contaminated-diet or sterile diet) in PDA medium, and identification of *F. verticillioides* tagged with the red fluorescent protein (*Fv:DsRed*) by microscopy. **b** Inoculation of macerated eggs of *D. saccharalis* (eggs from genitors fed on *F. verticillioides* contaminated-diet or sterile diet) in PDA medium, and identification of *Fv:DsRed* by microscopy. The bars represent 50 µm.


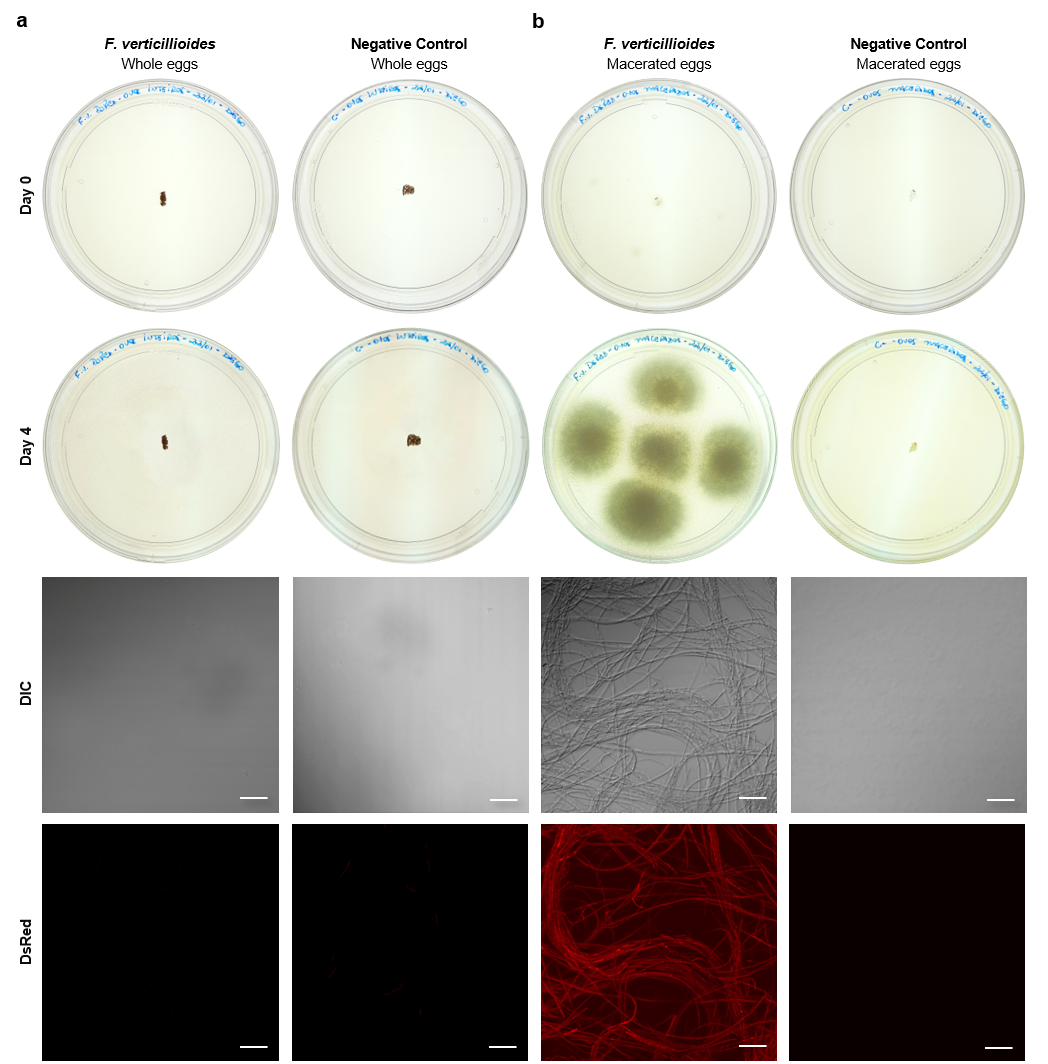


**Supplementary Fig. S9 *Fusarium verticillioides* transmission to plants.** **a** Experimental design to study the *F. verticillioides* transmission in sugarcane plants. The plants were inoculated for 72 h with third-instar offspring caterpillars from contaminated insects (genitors fed on a *Fv:DsRed*-colonized diet) and non-contaminated insects (genitors fed on a sterile diet). The samples were collected after 15 days of incubation. **b** The samples (from 2 to 5 cm around the inoculation area) from each plant were deposited on potato dextrose medium for seven days. **c** Identification of *Fv:DsRed* in plants by fluorescence. The bars represent 50 µm.


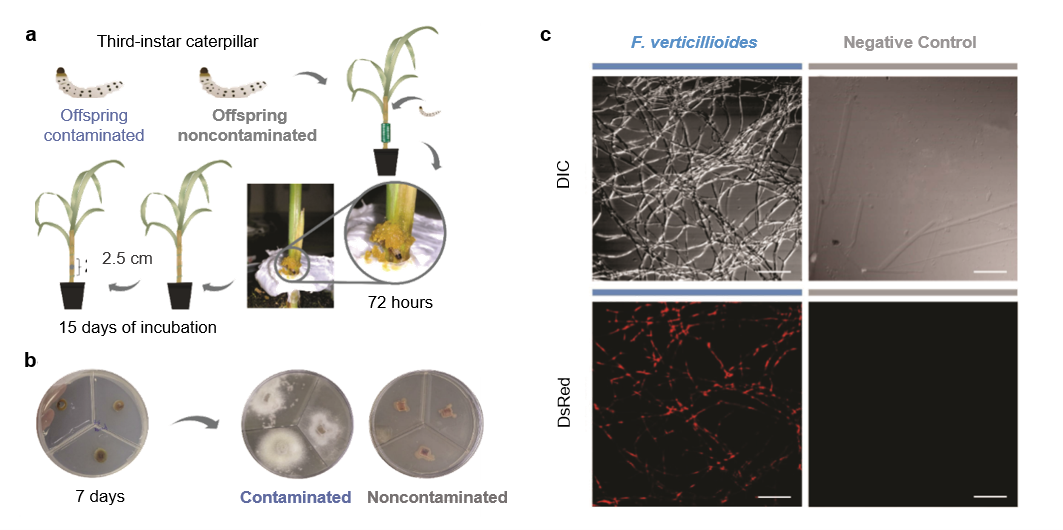


**Supplementary Tables S1-S3**

**Supplementary Table S1** Volatile organic compounds (VOCs) released by *Fusarium verticillioides* and *Aspergillus nidulans.* Mean amounts (± SEs in ng) of VOCs released by *A. nidulans* (negative control) and *F. verticillioides* cultivated in vitro during 8 hours of headspace collection (n = 6).

| Peak No. | Compound | RT (min) | Retention Index | *A. nidulans* | *F. verticillioides* | *p* |
| --- | --- | --- | --- | --- | --- | --- |
| 1 | 1-octen-3-ol* | 12.0 | 966 | 712.82 ± 178.44 | 8.63 ± 0.74 | 0.003 |
| 2 | Unknown 1 | 13.7 | 992 | 23.67 ± 6.45 | 0.0 ± 0.0 | 0.002 |
| 3 | Acoradiene | 26.4 | 1452 | 0.0 ± 0.0 | 36.05 ± 4.10 | 0.002 |
| 4 | Unknown 2 | 26.6 | 1461 | 0.0 ± 0.0 | 51.87 ± 9.47 | < 0.001 |
| 5 | Unknown 3 | 29.5 | 1593 | 0.0 ± 0.0 | 30.02 ± 2.65 | 0.002 |
| 6 | Acorenol | 29.7 | 1609 | 0.0 ± 0.0 | 556.48 ± 36.73 | 0.002 |

*p* < 0.05 indicates a significant difference between treatments according to the Kruskal-Wallis nonparametric test/parametric *t*-test.

*Compound identification based on retention index, MS library and synthetic standard

Compounds without * were identified based on retention index and MS library

**Supplementary Table S2** Volatile organic compounds (VOCs) released by *Fusarium verticillioides*-infected sugarcane. Mean amounts (± SEs in ng) of VOCs released by *F. verticillioides*-infected sugarcane and mock plants during 12 hours of headspace collection (n = 6).

| Peak No. | Compound | RT (min) | Retention Index | Mock | *F. verticillioides* | *p* |
| --- | --- | --- | --- | --- | --- | --- |
| 1 | Benzaldehyde * | 12.4 | 929 | 13.8 ± 13.3 | 65.4 ± 84.8 | 0.2 |
| 2 | 3.5.5-Trimethyl-1-hexene | 13.4 | 960 | 1.6 ± 1.3 | 236.3 ± 360.2 | 0.01 |
| 3 | 1-Octen-3-ol * | 13.7 | 968 | 18.2 ± 20.7 | 258.2 ± 143 | 0.01 |
| 4 | 3-Hexenyl-acetate * | 14.6 | 991 | 126.4 ± 187.7 | 3.2 ± 0.6 | 0.5 |
| 5 | 2-Octen-1-ol * | 16.7 | 1047 | 0.0 ± 0.0 | 16.7 ± 5.4 | 0.003 |
| 6 | Phenethyl alcohol * | 17.3 | 1079 | 0.0 ± 0.0 | 12.3 ± 4.1 | 0.003 |
| 7 | Unknown 4 | 30.6 | 1630 | 0.0 ± 0.0 | 86.3 ± 72.4 | 0.003 |
| 8 | Acorenol | 31.5 | 1659 | 0.0 ± 0.0 | 33.2 ± 7.2 | < 0.01 |
| 9 | 6.10.14-Trimethyl-2-pentadecanone | 32.2 | 1833 | 136.4 ± 81.7 | 143.8 ± 68.1 | 0.8 |
| 10 | Unknown 5 | 33.0 | 1880 | 298.74 ± 215.5 | 393.8 ± 251.4 | 0.5 |

*p* < 0.05 indicates a significant difference between treatments according to the Kruskal-Wallis nonparametric test/parametric *t*-test.

*Compound identification based on retention index, MS library and synthetic standard

Compounds without * were identified based on retention index and MS library

**Supplementary Table S3** Biological parameters (mean ± SE) of *Diatraea saccharalis* fed a *Fusarium verticillioides* diet and a control sterile diet (temperature 26 ± 4°C, relative humidity 60 ± 10% and 12 h of light).

|  | R_0_^*^ | T |  | r_m_ | ƛ |
| --- | --- | --- | --- | --- | --- |
| Control | 119.02 ± 20.26 | 47.85 ± 2.74 |  | 0.09 ± 0.0056 | 1.10 ± 0.0062 |
| *F. verticillioides* | 66.19 ± 15.37 | 51.11 ± 3.78 |  | 0.08 ± 0.0074 | 1.08 ± 0.0081 |

*Means differ significantly (t-test *p* < 0.05).

Ro=∑(l_x_m_x_); number of female eggs per female per generation, where l_x_ = the proportion of mated females alive at time x; and m_x_ = age-specific fecundity multiplied by the respective sex ratio; T = ∑(x l_x_m_x_)/∑(l_x_m_x_); the mean generation time; r_m_ = Ln Ro/T, the intrinsic rate of increase, where Ln = Neperian logarithm; ƛ = e^rm^, the finite rate of increase, where e = Euler-Lotka formula.

**Supplementary Video 1 Olfactory choice assay.** Ten caterpillars of *Diatraea saccharalis* (third-instar) were added into the central region of Petri dishes adapted with tubes containing sterile diet on one side (C) and *Fusarium verticillioides*-colonized diet on the opposite side (T). The video was recorded for 5 h and accelerated using Windows Movie Maker software.
